# A Novel Embedded Side Information Transmission Scheme Based on Polar Code for Peak-to-Average Power Ratio Reduction in Underwater Acoustic OFDM Communication System

**DOI:** 10.3390/s24227200

**Published:** 2024-11-10

**Authors:** Siyu Xing, Bo Wei, Yanting Yu, Xiaodong Gong

**Affiliations:** 1 Institute of Oceanographic Instrumentation, Qilu University of Technology (Shandong Academy of Sciences), Qingdao 266100, China; xingsiyu@qlu.edu.cn (S.X.); yuyanting@qlu.edu.cn (Y.Y.); gongxd@126.com (X.G.); 2 School of Ocean Technology Sciences, Qilu University of Technology (Shandong Academy of Sciences), Qingdao 266100, China; 3 Laoshan Laboratory, Qingdao 266237, China

**Keywords:** underwater acoustic communication (UAC), orthogonal frequency division multiplexing (OFDM), polar code construction, peak-to-average power ratio (PAPR) reduction, side information

## Abstract

In this paper, we proposed an embedded side information (SI) transmission scheme based on polar code construction for PAPR reduction using the PTS scheme in the underwater Acoustic (UWA) Orthogonal Frequency Division Multiplexing (OFDM) communication system. We use polar codes due to the ability of the arbitrarily designed code rate. Additionally, polar codes can be employed to establish a nested code structure consisting of multiple subsets. The SI bits can be embedded in a polar codeword by exploiting these features. Thus, the approach does not occupy existing data rates or cause additional loss in data transmission rates. At the same time, it embeds m-sequence into the polar code as an indicator vector for the blind SI detector, which makes the blind SI detector able to autonomously discriminate SI at the receiver. Simulation and tank experiment results indicate that the proposed embedded SI transmission scheme has the potential to significantly decrease the likelihood of whole-symbol error caused by SI errors. Meanwhile, the proposed PTS scheme eliminates the need to wait for the entire packet to be received before obtaining the SI, thereby preventing waste of data storage devices and ensuring real-time performance of the Underwater Acoustic Communication (UAC) OFDM system. This achieves symbol-level real-time calculation for the system.

## 1. Introduction

The environment of the underwater acoustic (UWA) channel is highly complex, making it the most difficult wireless communication channel. It exhibits time, frequency, space-selective fading, strong multipath, significant Doppler effects, high noise levels, and other intricate random transmission characteristics, resulting in underwater communication being significantly more complex than wireless communication in terms of reliability and high data rates [1,2,3]. Orthogonal frequency division multiplexing (OFDM) technology, with high spectral efficiency and excellent resistance to inter-symbol interference (ISI), can largely overcome the issues caused by the time-varying characteristics of UWA channels. Therefore, OFDM has been widely employed in the UWA communication systems [4,5,6].

Adopting the multi-carrier modulation scheme leads to in-phase superposition between subcarriers, resulting in a high peak-to-average power ratio (PAPR) in the UWA OFDM communication systems. In this case, if the linear dynamic range of some modules in the system (such as power amplifiers) is not sufficiently large, it will distort the transmitted signal, resulting in nonlinear distortion and the destruction of the orthogonality of subcarriers. High PAPR increases the demand for hardware and reduces the energy utilization rate [7]. Therefore, it is essential to reduce the PAPR. Currently, the PAPR reduction techniques are mainly classified into three categories: signal pre-distortion (clipping), coding, and probabilistic (scrambling) techniques. Among them, coding techniques have relatively limited applications due to the constraints of selectable codewords. Although signal pre-distortion techniques have simple principles and implementation methods, their aggressive clipping of signals can lead to significant losses in the bit error rate (BER) performance of the UWA OFDM systems and even fail to meet the requirements for reliable communication. A considerable amount of research aims to reduce the impact of nonlinear signal variations on the system’s BER performance, such as combining different signal pre-distortion techniques or signal pre-distortion with probabilistic techniques. However, combined algorithms do not merely incorporate the advantages but also simultaneously amplify the disadvantages [8,9].

The two promising probabilistic techniques are SLM [10] and Partial Transmit Sequence (PTS) [11]. These techniques optimize the OFDM symbol through phase scrambling, reducing the probability of maximum peak occurrence and effectively reducing the PAPR. However, multiple inverse fast Fourier transform (IFFT) operations are required to choose the OFDM symbol with the minimum PAPR, which suffers from an additional computational complexity that increases exponentially as the number of scrambling sequences increases. Researchers have proposed improved algorithms to balance the relationship between the PAPR reduction performance and the computational complexity [12,13,14,15]. A simplified PTS scheme is proposed in [12], in which computational complexity is significantly reduced compared to the scheme in [11], with a slightly degraded PAPR reduction performance. This scheme is referred to as the Conventional PTS (C-PTS) scheme in this paper. Setting a threshold for the PTS algorithm beforehand is also a method to reduce computational complexity. The iteration stops when the PAPR value of the current OFDM symbol is detected to be less than the threshold value. The reduction in computational complexity is also achieved at the cost of partially sacrificing PAPR reduction performance [13]. Reference [14] proposed a pseudo-optimal PTS scheme to reduce the computational complexity and improve the PAPR reduction performance by randomly selecting phase rotation vectors.

However, the other drawback of the SLM and PTS techniques is that they transmit several side information (SI) bits to recover the transmitted data accurately at the receiver. Thus, the overall BER performance heavily relies on the correctness of SI. Any error in SI transmission or detection can lead to a catastrophic BER for the entire system. To ensure the absolute correctness of SI, a particular encoding scheme or repeated SI sending multiple times is needed, which will require a massive amount of redundant data transmission, negatively impacting the communication efficiency and utilization of the communication bandwidth. Moreover, the SI data are usually transmitted after all other signals, which can also create storage pressure on the receiver and potentially compromise the real-time performance of the communication system.

Various phase rotation schemes, including concentric circle mapping, extended constellation, and cyclical shifting, are proposed to reduce the complexity and eliminate the need for separated SI transmission [16,17,18,19,20]. Joo H. S. proposed a blind SI detector for the SLM scheme based on the cyclic shift method, which alleviates the pressure of computational complexity but does not significantly improve the BER performance [17]. Reference [18] proposed a selective filtering technique in the time domain, which can select the transmit signal with the minimum PAPR in the time domain without additional IFFT operations. Meanwhile, the impact of time-domain filtering is treated as part of the multipath channel fading response to avoid SI transmission. However, to ensure the accuracy of SI, more pilot subcarrier allocation and a longer cyclic prefix are required. In Reference [19], the SI was linked to specific locations within the data block, and an SI detection block was utilized to identify the positions of the extended symbols. Reference [20] proposed a blind SI detection scheme based on minimum residual and minimum variance, considering the characteristics of underwater acoustic channels, which achieved autonomous identification and detection of SI information. Hussain I. M. proposed a method of embedding SI into data subcarriers before OFDM modulation [21]. Reference [22] assigned identifiable phase offsets to each rotating vector and incorporated the SI-identifying rotating vectors into the alternative signal sequences. These SI-embedded approaches can alleviate storage pressure on the receiving end, but the embedded SI reduces spectrum utilization and makes it challenging to ensure transmission accuracy.

Channel coding techniques are adopted in UWA communication systems to enhance reliability. Polar codes, invented by Erdal Arikan [23], have become an active research topic due to the promising results in increasing capacity. Polar code has been widely used and proven to perform better than the other coding schemes [24,25,26,27,28]. Furthermore, the polar code rate can be arbitrarily designed, making it more adaptable to different application scenarios [4,29,30]. The implementation of polar codes in the UWA OFDM communication system has demonstrated significant enhancements in the performance of underwater acoustic communication. This is due to their ability to mitigate the frequency selective fading that arises from the multipath effect in time-invariant and quasi-static channel conditions [4,6,28].

Additionally, the polar code can generate a nested code structure that is segmented into multiple subsets, each containing information bits and designated as the codebook for various users [31].

This paper proposes an embedded SI transmission scheme based on the polar code construction for PAPR reduction using the PTS scheme in the UWA OFDM communication system. This paper exploits the characteristics of polar codes, allowing for arbitrary code rate design. Additionally, the polar code can be employed to establish a nested code structure consisting of multiple subsets. The SI bits can be embedded in a polar codeword by exploiting these features. Thus, the approach does not occupy existing data rates or causes additional loss in data transmission rates. At the same time, it utilizes the cross-correlation characteristic of the m-sequence. It embeds m-sequence into the polar code construction and encoding process as an indicator vector for the blind SI detector. At the receiver, the blind SI detector can autonomously discriminate SI, providing perfect side information. The proposed embedded SI transmission scheme has significantly reduced the likelihood of whole-symbol errors caused by SI errors, as demonstrated by simulation and tank experiment results. Meanwhile, the proposed PTS scheme can avoid data storage device waste and ensure the real-time performance of the UAC OFDM system by eliminating the need to wait for the entire packet to be received before obtaining the SI, achieving symbol-level real-time calculation for the system.

The rest of the paper is organized as follows. Section 2 introduces the definition of the PAPR and the principle of the conventional PTS scheme. In Section 3, we present a novel embedded side information transmission scheme based on polar code, designed for the UWA OFDM communication system, including the generation of m-sequence, the encoder and the decoder of polar code for the proposed scheme. Section 4 provides the simulation and experimental results, while Section 5 presents the conclusions.

## 2. The Definition of the PAPR in the OFDM System and the Principle of the Conventional PTS Scheme

### 2.1. PAPR and PAPR Distribution

Normally, PAPR is defined as the ratio of the maximum power and the average power of the complex passband OFDM signal u(t).

(1) PAPRu˜(t)=max(Re(u˜(t)ej2πfct)2)ERe(u˜(t)ej2πfct)2=max(u(t)2)Eu(t)2,

In Equation (1), u(t) represents the complex passband signal, whilst $\tilde{u}(t)$ denotes the complex baseband signal. $E\left\{\cdot \right\}$ denotes the mathematical expectation, and max(·) means finding the maximum value of ·.

The complementary cumulative distribution function (CCDF) is used to describe the PAPR distribution of OFDM symbols. The formula for CCDF is provided in Equation (2).

(2) $${\tilde{F}}_{Z_{\max}}\left(z\right)=P\left(Z_{\max}>z\right)=1-P\left(Z_{\max}\leq z\right)=1-{\left(1-e^{-z^{2}}\right)}^{N},(z\geq 0),$$

### 2.2. The Principle and Side Information Transmission Scheme of Conventional PTS Scheme

**Figure 1:**
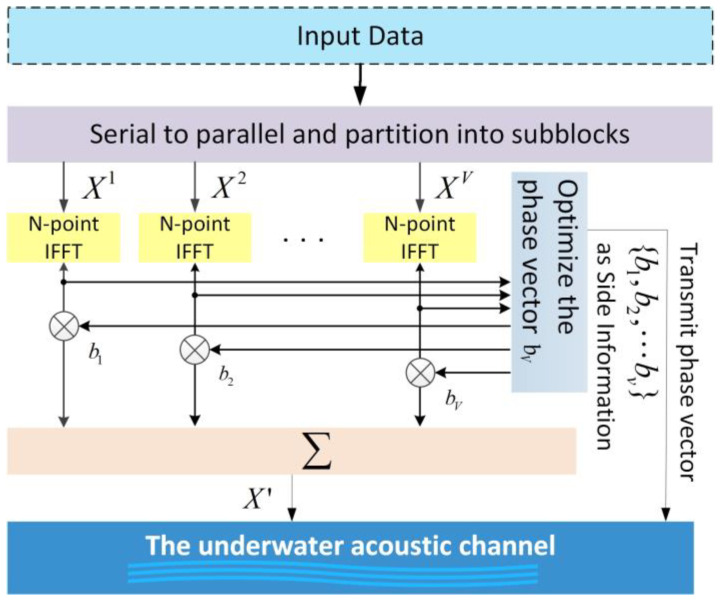
The framework of the C-PTS technique.

The C-PTS scheme first divided the input data block into disjoint subblocks and then combined them after applying phase rotation to each subblock. Finally, optimization of the phase vector was adopted to minimize the PAPR of the OFDM symbol. The framework of the C-PTS technique is given in Figure 1.

The input data block $X={\left[X_{0},X_{1},\cdots ,X_{N-1}\right]}^{T}$ is partitioned into V disjoint subblocks as follows:

(3) $$X={\left[X^{0},X^{1},X^{2},\cdots X^{V-1}\right]}^{T},$$

where $X^{v}$ denotes the subblocks with equal size. Each subblock multiplies the corresponding complex phase factor $b_{v}=e^{j\phi_{v}},v=1,2,\ldots ,V$ independently and then combines all the subblocks to obtain

(4) $$X^{\prime }=\sum_{v=1}^{V}b_{v}X_{v},$$

Subsequently, taking its IFFT to obtain the OFDM symbol in time domain

(5) $$x=\mathrm{IFFT}\{\sum_{v=1}^{V}b_{v}X_{v}\}=\sum_{v=1}^{V}b_{v}•\mathrm{IFFT}\{X_{v}\}=\sum_{v=1}^{V}b_{v}x_{v},$$

The phase vector $b_{v}$ should be selected in such a way as to minimize the PAPR of the OFDM symbols, as demonstrated by Equation (6).

(6) $$\{{\tilde{b}}_{1},{\tilde{b}}_{2},\cdots ,{\tilde{b}}_{v}\}=\arg\min\left(\underset{n=0,1,\cdots N-1}{\max}{\left|\sum_{v=1}^{V}b_{v}x_{v}\right|}^{2}\right),$$

Normally, any value within the range [0,2π) can be chosen as $b_{v}$ to ensure minimum PAPR. However, the search complexity increases exponentially with the number of subblocks, making it a heavy burden for the communication system. Therefore, a simplified scheme is fixed $b_{v}$ in the set {1,−1} [23], which slightly sacrifices PAPR reduction performance. Another disadvantage of the C-PTS scheme is SI transmission, which causes data rate loss.

Moreover, the overall system bit error rate (BER) performance depends on the accuracy of the side information. A particular encoding scheme is generally required to ensure the absolute correctness of SI, or multiple repetitions of transmission are needed to ensure that the receiving end can accurately recover the signal. Such a massive amount of redundant data transmission wastes the already very tight underwater acoustic communication bandwidth resources, reducing the efficiency of the UWA OFDM communication system. Meanwhile, symbols used to transmit SI are usually attached to the end of the original OFDM signal for transmission, affecting the system’s real-time performance.

## 3. The Principle of the Embedded Side Information Transmission Scheme Based on Polar Code

### 3.1. The Polar Code Construction of the Nested Code Structure and the Encoder for the Proposed Embedded SI Transmission Scheme

(N,K,F) can represent a specific polar code. The code length N usually needs to be at any finite length of $2^{n}$. K represents the length of the information bits in a codeword. F is a set of frozen bit locations containing N−K integer indices. The polar code rate (CR) can be defined as $C_{rate}=K/N$.

When there is more than one set of input data in a polar code word, there is a corresponding relationship between each set of input data and the subset of channels [4]. The polar codeword with the nested code structure can be generated as:

(7) $$x=u_{data1}G_{N}(F_{data1}^{C})⊕u_{data2}G_{N}(F_{data2}^{C})⊕\cdots ⊕u_{datai}G_{N}(F_{datai}^{C})⊕u_{F}G_{N}(F),$$

where $u_{datai}$ is a vector information bit, representing the $i^{th}$ input data set. $G_{N}$ is the generator matrix, and $F^{C}$ is the complementary set of F, known as the information bit locations. $F^{C}$ can be divided into disjoint subsets $F_{datai}^{C}$ allocated to different encoded data information bit sets at a specific code rate. The matrices $G_{N}(F_{datai}^{C})$ are part of the matrix $G_{N}$, formed by the rows corresponding to the information bit locations in the set $F_{datai}^{C}$. $G_{N}(F)$ is determined by the frozen bit locations F and $G_{N}$.

Since polar code can be utilized to create a nested code structure, the construction of the polar code for the proposed embedded SI transmission scheme can be achieved as follows.

Define N as the polar code length, $K_{SI}$ as the embedded SI indicator vector length, and also the m-sequence length. $K_{data}$ represents the data bits length in a codeword. Divide the information bit locations $F^{C}$ into two disjoint subsets $F_{SI}^{C}$ and $F_{data}^{C}$, satisfying $F_{SI}^{C}\cup F_{data}^{C}=F^{C}$. These subsets are used to transmit the m-sequence $u_{SI}=(u_{1},u_{2},\ldots ,u_{K_{SI}})$, which is used as the indicator vectors for the blind detector of SI, and the data $u_{data}=(u_{1}^{\prime },u_{2}^{\prime },\ldots ,u_{k}^{\prime })$, respectively.

Typically, for the reliability measurement of polarized bit-channels, the Bhattacharyya parameter recursive evolution algorithm is used to obtain the $F^{C}$, the set of information bit locations. The Bhattacharyya parameter is defined as Equation (8), and Log-domain calculations are necessary to prevent underflow and overflow.

(8) $$B(W)\overset{\Delta }{=}\sum_{y\in Y}\sqrt{W(y|0)W(y|1)},$$

The logarithm of the bit-channel Bhattacharyya parameter $\mathrm{lg}[B(W_{N}^{i})]$ should be arranged in ascending order, and an array flag is constructed to record the index of the bit-channel. In contrast, the other array is set to record the corresponding $\mathrm{lg}[B(W_{N}^{i})]$.

Then, the top $K=K_{SI}+K_{data}$ values in the array flag are arranged to the information bits indexes, including the m-sequence and the transmitted data. The rest N−K values are for the frozen bits. σ represents the threshold of the $\mathrm{lg}[B(W_{N}^{i})]$ with the code rate $C_{rate}=K/N$.

Finally, the position of SI bit locations $F_{SI}^{C}$ and the transmitted data location $F_{data}^{C}$ are determined. There are two types of the $F_{SI}^{C}$ and $F_{data}^{C}$ selection. One is to assign the first $K_{SI}$ bits in $F^{C}$ to $F_{SI}^{C}$ and the next $K_{data}$ bits to $F_{data}^{C}$. The other is to allocate the first $K_{data}$ bits to $F_{data}^{C}$ and the next $K_{SI}$ bits to $F_{SI}^{C}$. The simulation analysis will present a performance comparison of different combinations of $F_{SI}^{C}$ and $F_{data}^{C}$.

**Figure 2:**
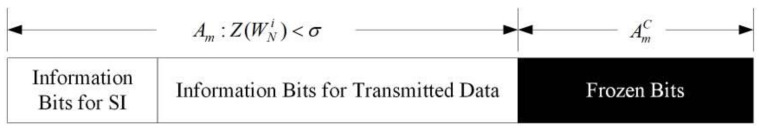
The polar code construction diagram designed for the proposed embedded SI scheme.

Thus, the polar code construction diagram designed for the proposed embedded SI is presented in Figure 2.

**Figure 3:**
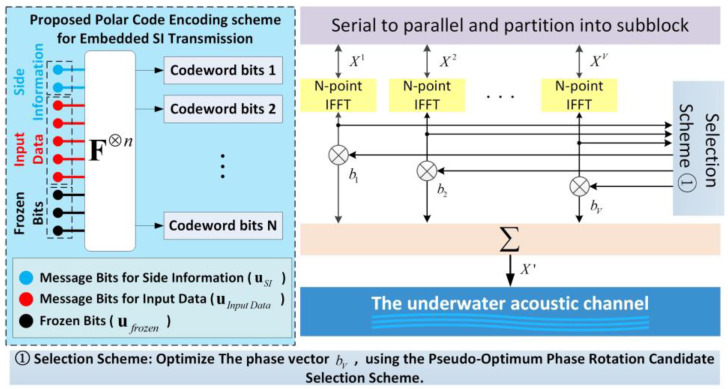
The framework of the embedded SI transmission based on polar code construction for PAPR reduction using the PTS scheme in UWA OFDM communication system. The pseudo-optimum phase rotation candidate selection scheme in this Figure is proposed in Ref. [14].

When $F^{C}$ and its two disjoint subsets $F_{SI}^{C}$ and $F_{data}^{C}$ are determined, the m-sequence and the transmission data can be encoded. The encoded polar codeword is given as:

(9) $$x_{1}^{N}=u_{SI}G_{N}(F_{SI}^{C})⊕u_{data}G_{N}(F_{data}^{C})⊕u_{F}G_{N}(F),$$

where matrices $G_{N}(F_{SI}^{C})$ and $G_{N}(F_{data}^{C})$ are both parts of the matrix $G_{N}$, formed by the rows in $G_{N}$ that correspond to the SI bits locations $F_{SI}^{C}$ and the transmitted data location $F_{data}^{C}$, respectively. The framework of the embedded SI transmission based on polar code construction for PAPR reduction using the PTS scheme in the UWA OFDM communication system is illustrated in Figure 3. In this diagram, the side information and data information are located in different information bit locations, and the frozen bits are located in the noisy bit-channels, transmitted as a frozen bit sequence $u_{F}$ of all-zero vectors. Since the SI indicator vector used for blind detection is already embedded in the transmitted data encoded by the polar code, adding an indicator vector is unnecessary after optimizing the weighted phase factor vector $b_{V}$. The optimization process of $b_{V}$ adopted in this paper is described in [14].

The encoded polar codeword $x_{1}^{N}$ goes through constellation mapping, OFDM modulation, and the PAPR reduction in sequence. Then, the OFDM symbol with the lowest PAPR value is selected as the transmitted signal $x^{\prime }$.

### 3.2. The Decoder for the Proposed Scheme and the Principle of the Blind SI Detector

The core idea of the proposed SI blind detector is to use the embedded m-sequence as the indicator vector. By judging the cross-correlation values between the decoded M sets of m-sequences and the transmitted m-sequence, autonomous discrimination of SI can be achieved.

The received signal in the frequency domain can be given as

(10) Y(k)=X(k)H(k)+W(k),

where H(k) and W(k) denote the frequency response of the UWA channel and the white Gaussian noise in the frequency domain, respectively. Define ${\hat{H}}_{P}(k)$ as the sampling value of the estimation of the underwater acoustic channel frequency response $\hat{H}(k)$. Using the received and transmitted comb pilot signal, ${\hat{H}}_{P}(k)$ can be obtained by ${\hat{H}}_{P}(k)=Y_{P}/X_{P}$ and then gained $\hat{H}(k)$ through interpolation.

After applying the channel equalization scheme, the data with phase rotation $X^{\prime }(k)$ perform inverse phase rotation on the M sets of different phase weighting factors $b_{v}^{m}$, respectively, extracting the data on the data subcarriers to obtain $X_{D}^{m}(k)$. After constellation de-mapping, the polar decoding needs to be conducted, and the bit decision rule of the Successive Cancellation (SC) decoder can be given as Equation (11).

(11) $${\hat{u}}_{i}^{m}=\left\{\begin{array}{l} 0,\;\;i\in F\\ 0,\;\;i\in F^{C}\;\&\;L_{1,j}\geq 0\\ 1,\;else \end{array}\right.,$$

Additional steps are required to complete the decoding step for polar codes embedded with m-sequences. The extra step is to determine whether the bit-channels in the decoded output belong to the estimated SI data ${\hat{u}}_{SI}^{m}$ or the estimated transmitted data ${\hat{u}}_{data}^{m}$. The decision rule is expressed in Equation (12).

(12) $$\left\{\begin{array}{l} {\hat{u}}_{SI}^{m}\;\;\;={\hat{u}}_{i}^{m},\;\;\;i\in F_{SI}^{C}\\ {\hat{u}}_{data}^{m}={\hat{u}}_{i}^{m},\;\;i\in F_{data}^{C} \end{array}\right.,$$

The estimated SI data ${\hat{u}}_{SI}^{m}$, representing the estimated value of the M sets of m-sequences, consist entirely of elements in this sequence belonging to the set $\left\{0,1\right\}$. To enhance the difference in the cross-correlation values between ${\hat{u}}_{SI}^{m}$ and the embedded m-sequences $u_{SI}$ at the transmitter, all the ${\hat{u}}_{SI}^{m}$ and $u_{SI}$ are converted into bipolar codes, i.e., the elements belong to the set $\left\{-1,1\right\}$. Then, the cross-correlation value $R\left({\hat{u}}_{SI}^{m},u_{SI}\right)$ between ${\hat{u}}_{SI}^{m}$ and the embedded m-sequences $u_{SI}$ at the transmitting end is calculated. Finally, the ${\hat{u}}_{data}^{m}$ corresponding to the maximum cross-correlation value $R\left({\hat{u}}_{SI}^{m},u_{SI}\right)$ is selected as the final decoded output.

The discrimination of the cross-correlation value $R\left({\hat{u}}_{SI}^{m},u_{SI}\right)$ between ${\hat{u}}_{SI}^{m}$ and $u_{SI}$ is related to the length of the m-sequences. Due to the randomness in constructing the candidate weighted phase factor vectors at the transmitter and the randomness of decoding errors at the receiver, the data obtained from inverse phase rotation with the wrong phase weighting factors still have a probability of recovering the complete m-sequences correctly. However, the probability will be significantly reduced with the increase in the length of the m-sequences. Therefore, when the amount of information that a single OFDM symbol can carry is the same as or similar to the length of the polar code, the reliability of the SI blind detection algorithm at the receiver can be ensured by appropriately increasing the length of the m-sequences.

When the amount of information that a single OFDM symbol can carry is an integer multiple of the length of the polar code, for the bit-channels where the side information is located, it can be considered that they have experienced different frequency selective fading, which can be regarded as frequency diversity. In this case, diversity combining methods can be used to obtain diversity gains and improve the discrimination accuracy of the cross-correlation values $R\left({\hat{u}}_{SI}^{m},u_{SI}\right)$. Typical diversity combining techniques include maximum ratio combining, selection combining, and equal gain combining. For the proposed scheme in this section, maximum ratio combining is unsuitable. Although different subcarrier frequencies will experience different frequency selective fading, the calculation of the signal-to-noise ratio (SNR) for subcarriers is not straightforward and simple, and it will increase the system’s complexity. Similarly, selection combining also requires the calculation of the SNR of subcarriers. Therefore, equal gain combining can be used to improve the discrimination of the cross-correlation value $R\left({\hat{u}}_{SI}^{m},u_{SI}\right)$ by simply summing the $R\left({\hat{u}}_{SI}^{m},u_{SI}\right)$ obtained from decoding different codewords within the same OFDM symbol, which facilitates improving the accuracy of autonomous SI recognition.

## 4. The Results and Discussion of the Simulation and the Tank Experiment

### 4.1. Simulation Results and the Discussion

The main parameters of the UAC OFDM system are given in Table 1. The number of total subcarriers in one OFDM symbol is 1024, the length of the polar code is N=512, and the number of bits per subcarrier is 2. At this time, the actual information in each OFDM symbol is equivalent to three polar codewords. The traditional PTS (C-PTS) scheme, which requires the transmission of side information (SI), is used as a control group. It employs a polar code with data information bits K=256. The SI information and data information bits use precisely the same encoding and mapping methods, and the OFDM symbols carrying SI information are transmitted after the data OFDM symbols. In the PTS algorithm, the number of subblocks is V=4, so the total number of candidate weighted phase factor vectors is $M=2^{V-1}=8$. Although the embedded m-sequence does not reduce the number of sub-channels allocated to information bits, it may slightly increase the polar code rate. It is possible to determine the length of the m-sequence according to the code length of the polar code, with the premise of having a small overall BER impact on the information bits. The length of the m-sequence occupies less than 10% of the information bit length in the polar code. The length of the polar code adopted in the proposed scheme is N=512, and the number of side information bits within a polar code, which is also the length of the m-sequence, is 15. The data information bits and the frozen bits within a codeword are 256 and 241, respectively.

**Figure 4:**
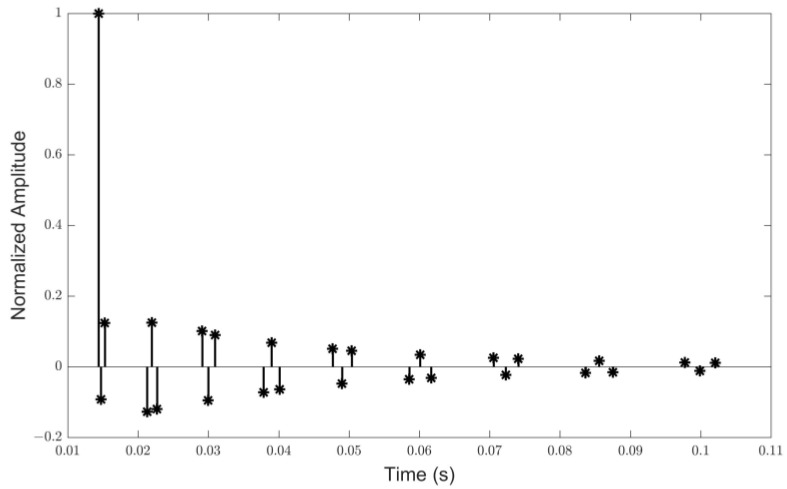
The channel impulse response of the UWA simulation channel.

Channel simulation software generates the sparse shallow water acoustic channel adopted in the simulations. The average depth of the sea is set to 55 m. The transducer and the hydrophone are located 8 m under the water surface, and the distance between them is about 3 km. The velocity gradient distribution is set as a surface channel. The channel impulse response (CIR) is illustrated in Figure 4.

**Figure 5:**
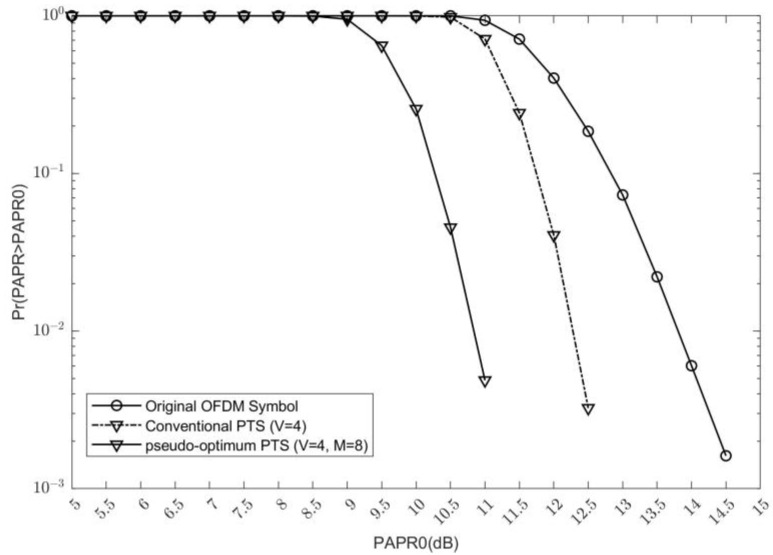
The PAPR reduction performance comparison of the original OFDM signal, the C-PTS scheme and the pseudo-optimum PTS scheme.

The proposed scheme adopts the pseudo-optimum PTS scheme given in Reference [14], which reduces the computational complexity and modifies the PAPR reduction performance. The PAPR reduction performance is given in Figure 5. The number of subblocks and phase rotation candidates in the pseudo-optimum PTS scheme are the same as in the C-PTS scheme. In Figure 5, it can be seen that the pseudo-optimum PTS scheme has about 3.5 dB and 1.5 dB gains when compared with the original OFDM symbol and the C-PTS scheme, respectively.

In the subsequent part of this section, we mainly focus on the overall BER performance comparison between the C-PTS and the proposed scheme in this paper.

**Figure 6:**
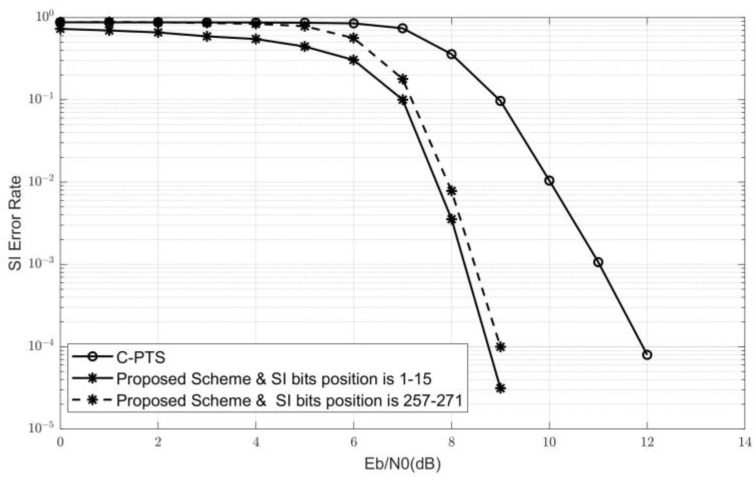
The SI error rate comparison among the different $F_{SI}^{C}$ positions.

Firstly, the SI error rate $P_{s}$ comparison among the side information bit locations $F_{SI}^{C}$ with different positions in $F^{C}$ is given in Figure 6. The SI bit position 1∼15 or 257∼271 indicates the relative position of the subset $F_{SI}^{C}$ in $F^{C}$, rather than the original index values of each bit-channel recorded in the flag array during the sorting process. SI bits position is 1∼15, which means that the SI bit locations set $F_{SI}^{C}$ is formed by the first $K_{SI}$ bit-channels with the smallest Bhattacharyya parameter pair values, which means the highest reliability among the K records of the information bit locations set $F^{C}$. Conversely, it means that the $K_{SI}$ bit-channels with the lowest reliability among the K records are selected to form $F_{SI}^{C}$.

The BER curve of the C-PTS scheme with known SI in Figure 6 is used as a control group. From the two SI error rate curves with asterisks in Figure 6, it can be seen that the SI accuracy utilizing the correlation of m-sequences for discrimination significantly improved by about 3 dB compared to the control group. The SI embedded in bit-channels with higher reliability has an improvement of about 0.3 dB compared to bit-channels with lower reliability. Based on the previous analysis, the SI error rate is the main factor affecting the overall system BER performance. Therefore, when constructing a polar code with embedded SI, it is necessary to select the $K_{SI}$ bit-channels with the highest reliability in $F^{C}$ as $F_{SI}^{C}$.

**Figure 7:**
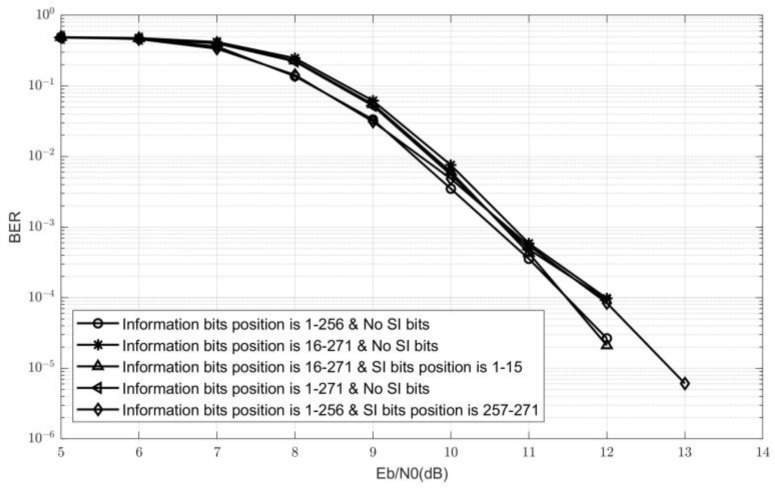
The BER performance comparison among the different $F_{data}^{C}$.

Secondly, assuming that the side information is entirely correct, the overall BER performance comparison among the transmitted data bit locations $F_{data}^{C}$ with different positions in $F^{C}$ is given in Figure 7. No SI bits indicate that during the polar code construction, all bit-channels are frozen bits except for the information bits required for transmitting data.

The data bit position is 1∼256 with no SI bits, which indicates that the polar code construction scheme used in the C-PTS scheme has a code rate of $C_{c}=K/N$. The data position is 1∼271 with no SI bits, which means that the polar construction scheme with a code rate of $C_{s}=\left(K+K_{SI}\right)/N$ indicates that the information bit location of this polar code construction is $F_{SI}^{C}\cup F_{data}^{C}$. It can be concluded that the BER performance of these two cases only has a difference of about 0.5 dB when $7\mathrm{dB}<Eb/N_{0}<10\mathrm{dB}$, and the $Eb/N_{0}$ increasing difference value continues to shrink when $Eb/N_{0}>10\mathrm{dB}$. This is because the length of $K_{SI}$ is not very large, and the code rate difference is not significant.

When the data bit position is 16∼271, and the SI bit position is 1∼15, the $F_{SI}^{C}$ is formed with the bit-channels with higher reliability. As mentioned in the previous section, this embedded polar code construction scheme has the lowest SI error rate. The reason for selecting the polar code construction scheme with information bits 16∼271 and no SI bits is to compare the BER performance of the same number and location distribution of transmitted data bits with and without SI bits. As shown in Figure 7, after setting the SI bit positions to frozen bits, the BER performance of the data bits is slightly worse than that of the polar code construction scheme with SI bits when $Eb/N_{0}$ is high. This is because when constructing a polar code, the $K_{SI}$ bit-channels with the highest reliability are discarded, which can lead to a slight loss in BER performance for other data bits.

**Figure 8:**
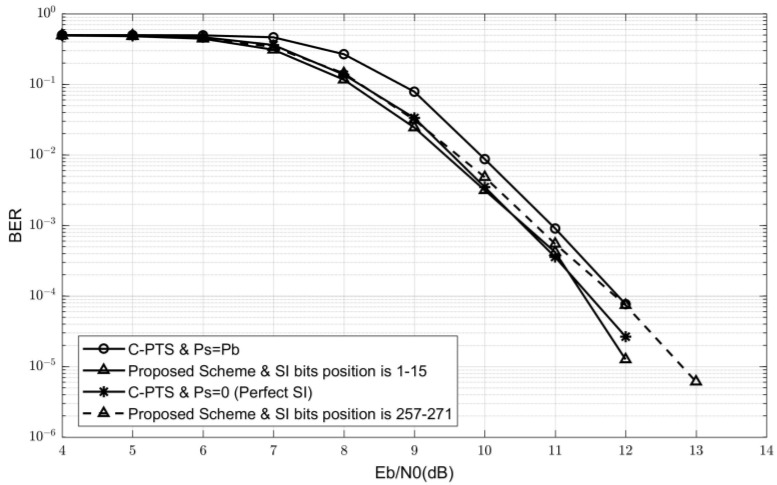
The overall BER performance comparison among different combinations of $F_{SI}^{C}$ and $F_{data}^{C}$.

Overall, although the BER performance of the polar code construction scheme with a data bit position of 1∼256 and SI bit position of 257∼271 is the worst, the maximum difference in BER between all polar code construction schemes is not more than 0.5 dB. Therefore, it can be inferred that the selection of different $F_{data}^{C}$ does not significantly impact the BER performance $P_{b}$. The SI error rate $P_{s}$ mainly determines the overall system BER performance. Therefore, the optimal construction scheme for embedding polar codes in SI should be that $F_{SI}^{C}$ represents the first $K_{SI}$ bits in $F^{C}$, and $F_{data}^{C}$ represents the next $K_{data}$ bits. The simulation results in Figure 8 also verify this conclusion.

Finally, the overall BER performance comparison among different combinations of $F_{SI}^{C}$ and $F_{data}^{C}$ is shown in Figure 8. Unlike the previous simulations, SI errors exist in these simulations. When the polar code construction scheme selects $F_{SI}^{C}$ as the first $K_{SI}$ bits in $F^{C}$ and $F_{data}^{C}$ as the next $K_{data}$ bits, the BER performance curve is basically consistent with the PTS scheme with perfect SI. It also has an improvement of about 0.5 dB compared to the C-PTS scheme. The BER performance of the SI-embedded polar code construction scheme, where the first $K_{data}$ bits are taken as $F_{data}^{C}$ and the subsequent $K_{SI}$ bits are $F_{SI}^{C}$, is better than the C-PTS scheme when $Eb/N_{0}$ is less than 12 dB. It is because $P_{s}$ is much smaller than $P_{b}$ at these points. As $Eb/N_{0}$ increases, both $P_{s}$ and $P_{b}$ of C-PTS tend to zero. The system BER P of this construction scheme then mainly depends on $P_{b}$. As shown in Figure 7, the $P_{b}$ performance of this construction scheme is somewhat worse than the C-PTS scheme. Therefore, its system BER performance is not good at high $Eb/N_{0}$ values. The simulation results further demonstrate that the optimal SI-embedded polar code construction scheme should involve the first $K_{SI}$ bits from $F^{C}$ for $F_{SI}^{C}$, and then using the subsequent $K_{data}$ bits for $F_{data}^{C}$, and this combination will be selected in subsequent pool experiments.

### 4.2. Tank Experimental Results and the Discussion

A tank experiment is conducted to verify the reliability of the proposed scheme. The practical dimensions of this pool are 45 m×6 m×5 m. High-frequency sound-absorbing materials surround the four walls of the tank, and the bottom is sandy, which can effectively simulate the multipath channel of underwater acoustic communication. The propagation characteristics of sound waves in the tank, including the distinction between direct waves and reflected waves, as well as their time intervals, are taken into consideration when locating the transmitter and the receiver. In this experiment, the transmitter and receiver were located at depths of 2.5 m and 2 m below the water surface, with a horizontal distance of approximately 8.35 m.

**Figure 9:**
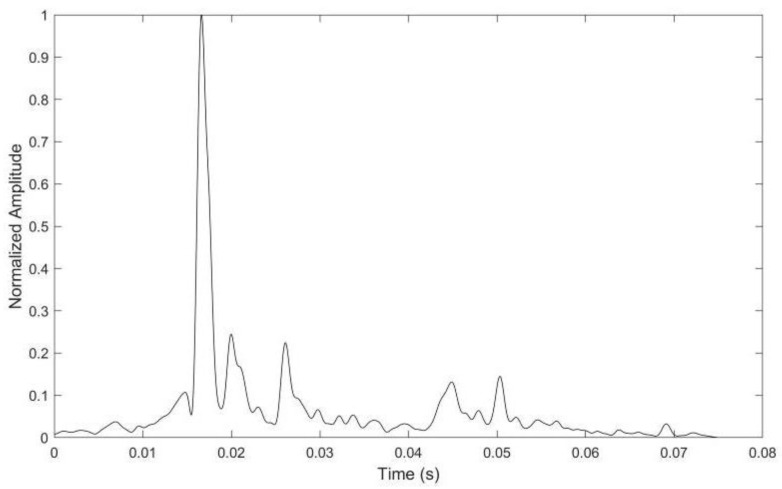
The real CIR of the tank experiment channel.

The real CIR of the tank channel is shown in Figure 9. The OFDM system parameters used in this experiment are the same as the simulation parameters in Table 1. The polar code construction scheme with embedded SI adopts the best scheme verified in the simulation. The tank experiment’s received signal-to-noise ratio (SNR) is approximately 10.98 dB.

**Figure 10:**
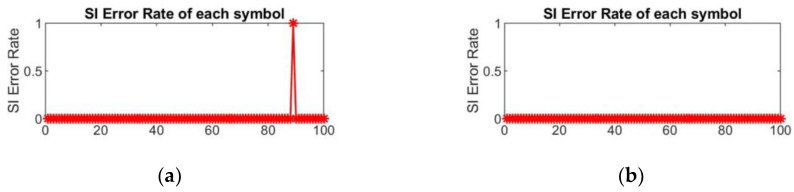

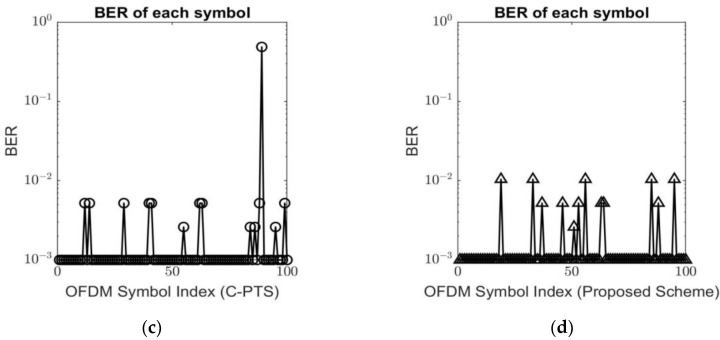
Tank experimental results comparison of the SI transmission between the C-PTS and the embedded SI transmission based on the polar code construction. (**a**) SI error rate of each symbol in C-PTS scheme; (**b**) SI error rate of each symbol in the proposed scheme; (**c**) BER of each symbol in C-PTS scheme; (**d**) BER of each symbol in the proposed scheme.

Figure 10 presents the SI error rate and the BER of the corresponding symbol for the first 100 OFDM symbols transmitted in the tank experiment. The number of information bits in each OFDM symbol is 756, so $\mathrm{BER}=10^{-3}$ indicates that there are no errors within the symbol. In Figure 10, (a) and (c) represent the SI error rate of each symbol and the system BER performance affected by SI errors in the C-PTS scheme, while (b) and (d) represent the SI error rate and system BER performance of the SI transmission algorithm based on the proposed embedded polar code scheme. It can be seen that the C-PTS algorithm experiences an SI misjudgment at the 89th OFDM symbol, with an SI error rate of 1. Correspondingly, there is a clear peak at the 89th symbol in the symbol error rate curve. On the other hand, the proposed scheme with a blind SI detector based on embedded polar code construction does not experience any SI errors, with only a few symbols having bit errors, resulting in better BER performance.

**Figure 11:**
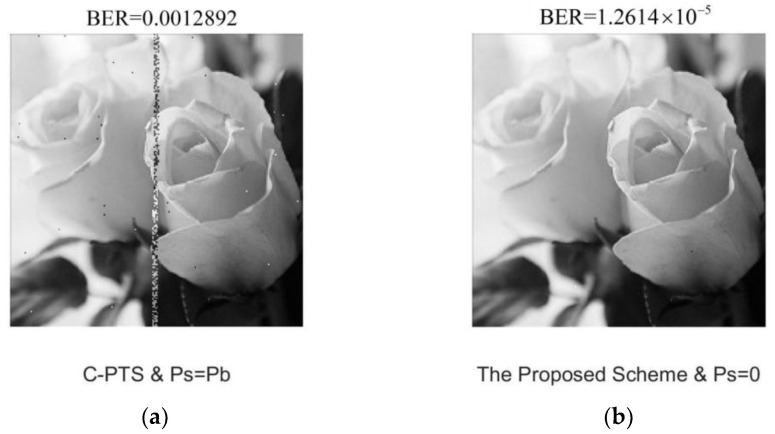
Comparison of transmitted figure results from the tank experiment. (**a**) The transmitted figure result of the C-PTS scheme; (**b**) the transmitted figure result of the proposed scheme.

Figure 11 compares the image transmission results in this tank experiment. The encoding and modulation method for SI information is the same as for image data information in the C-PTS scheme. The left image in Figure 11 shows the transmitted result of the UAC OFDM system using the C-PTS technique. Due to the SI error’s influence, a striped noise area exists, which makes the overall BER approximately two orders of magnitude higher than that of the proposed scheme using a blind SI detector (right image).

## 5. Conclusions

This paper proposed an embedded SI transmission scheme based on polar code construction for PAPR reduction using the PTS scheme in the UAC OFDM system. Due to the ability of the arbitrarily designed code rate and the nested code structure, the SI bits can be embedded in a polar codeword. Besides the data transmission code rate, it adds several additional information bits to transmit side information. Unlike other embedding methods, this approach does not occupy existing data rates and does not cause additional loss in data transmission rates. At the same time, it utilizes the cross-correlation characteristic of the m-sequence. It embeds m-sequence into the polar code construction and encoding process as an indicator vector for the blind SI detector. At the receiver, the blind SI detector can autonomously discriminate SI, providing perfect side information. The probability of whole-symbol error caused by SI errors can be significantly reduced. Meanwhile, the proposed PTS scheme eliminates the need to wait for the entire packet to be received before obtaining the SI, thereby preventing waste of data storage devices and ensuring real-time performance of the UAC OFDM system, achieving symbol-level real-time calculation for the system. The simulation and tank experiment results indicate that the proposed embedded SI transmission scheme based on polar code for the PTS scheme in the UAC OFDM system significantly guarantees the reliability of the overall BER performance.

## Figures and Tables

**Table 1:** Parameters of the UWA OFDM system.

Parameters	Value
Transmission Frequency Band	8–16 kHz
Sampling Frequency	48 kHz
Number of Subcarriers	1024
Subcarrier Bandwidth	7.81 Hz
Oversampling Rate	4
Number of Bits per Subcarrier	2(QPSK modulation)
Pilot Spacing	4
Subblock Number	4
Number of Complex Phase Factors	8
Polar Code Length	512
Data Information Bits within a Polar Code	256
Side Information Bits within a Polar Code	15
Frozen Bits within a Polar Code	241

## Data Availability

The data analyzed during the current study are available upon reasonable request from the corresponding author.
